# Supplementary material for: Embedded-AMP: A Multi-Thread Computational Method for the Systematic Identification of Antimicrobial Peptides Embedded in Proteome Sequences
Source: Antibiotics (Basel). 2023 Jan 10;12(1):139. doi: 10.3390/antibiotics12010139 (PMC9854971; doi:10.3390/antibiotics12010139)
Supplement: Supplementary file 1 [file antibiotics-12-00139-s001.zip › antibiotics-2022864-supplementary.pdf]

Table S1. Embedded AMP found in GAPDH in *Saccharomyces cerevisiae*

Experimental AMP	Protein ID	Predicted
**VSWYDNEYGYSTR**	G3P2_YEAST	Concatenated
**ISWYDNEYGYSAR**	G3P1_YEAST	No
**VSWYDNEYGYSTRV**	G3P2_YEAST	Concatenated
**ISWYDNEYGYSARV**	G3P1_YEAST	No
**VSWYDNEYGYSTRVV**	G3P2_YEAST	Concatenated
**FRVPTVDVSVVD**	G3P2_YEAST, G3P1_YEAST	Concatenated
**FRVPTVDVSVVDL**	G3P2_YEAST, G3P1_YEAST	Concatenated
**ISWYDNEYGYSAR**	G3P1_YEAST	No
**VSWYDNEYGYSTR**	G3P2_YEAST	Concatenated
**VSWYDNEYGYSTRV**	G3P2_YEAST	Concatenated
**LVSWYDNEYGYSTR**	G3P2_YEAST	Concatenated
**ISWYDNEYGYSARV**	G3P1_YEAST	No
**ISWYDNEYGYSAR**	G3P1_YEAST	No
**VSWYDNEYGYSTR**	G3P2_YEAST	Concatenated
**ISWYDNEYGYSARV**	G3P1_YEAST	No
**VSWYDNEYGYSTRV**	G3P2_YEAST	Concatenated

Experimental ID column refers to the peptide sequence that was experimentally identified as AMP from the glyceraldehyde 3-phosphate dehydrogenase protein in yeast [9]. Protein ID refers to the Uniprot accession ID of the protein that embeds the AMP identified. Predicted corresponds with the prediction as AMP by our method; there are 2 different classes of predictions: No, this AMP was not predicted as AMP by our method; Concatenated, this peptide sequence was concatenated to other AMP that were overlapping with it and predicted as AMP.

Table S2. Embedded AMP found in the proteome of *Saccharomyces cerevisiae*

Experimental AMP	Protein ID	Predicted
**PVGDQPPDIERQIK**	VPS1_YEAST	No
**PVGDQPPDIERQIKD**	VPS1_YEAST	No
**TEDPAKNFQPDTGRIE**	PYC1_YEAST	Embedded
**PVGDQPPDIERQIKDM**	VPS1_YEAST	No
**APPLPPSAPPPP**	VRP1_YEAST	No
**APPLPPTAPPPP**	VRP1_YEAST	No
**LPPPPPPSVATSVP**	VRP1_YEAST	No
**GAPAPPPPPPPPALGGSAPKP**	VRP1_YEAST	Exact
**TRPGDLPEKDF**	WTM1_YEAST	No
**IIDNAGKPGEILRT**	WTM1_YEAST	Exact
**IAAIGADLIDERIIDQ**	EF3A_YEAST	Embedded
**IAAIGADLIDERIIDQQ**	EF3A_YEAST	Embedded

**IVLDEPTNYLDRDSLGA**	EF3A_YEAST/EF3B_YEAST	Embedded
**AEPISSENGVLEPR**	YG3A_YEAST	No
**GGFGGPGGPGGQGFGRQGPQG**	YNU8_YEAST	Exact
**KEGDDAPESPDIHF**	YRB1_YEAST	No
**LEETKESLQNKGQEVKEQ**	ZEO1_YEAST	No

Experimental ID column refers to the peptide sequence that was experimentally identified as AMP [30]. Protein ID refers to the Uniprot accession ID of the protein that embeds the AMP identified. Predicted corresponds with the prediction as AMP by our method; there are 3 different classes of predictions: No, this AMP was not predicted as AMP by our method; Concatenated, this peptide sequence was concatenated to other AMP that were overlapping with it and predicted as AMP; Embedded, this peptide sequence was found as part of another larger AMP identified by our method.

Table S3. Peptides molecular descriptors

Index	Molecular descriptor name	Index	
**1**	Sequence length	**27**	Average hydrophobicity TOSSI2002 [50]
**2**	Phenylalanine composition	**28**	Distribution of "EALMQKRH" at 50% [51,52]
**3**	Net charge at pH 9.0	**29**	Distribution of "EALMQKRH" at 100% [51, 52]
**4**	Distribution of "MHKFRYW" at 50% [51, 52]	**30**	Distribution of "CLVIM" [53]
**5**	Methionine composition [54]	**31**	Distribution of "DE" at 0% [51, 52]
**6**	Distribution of "NVEQIL" at 75% [51, 52]	**32**	Distribution of "FWY" [53]
**7**	Glutamine composition [54]	**33**	Distribution of "KR" at 100% [51, 52]

Index	Molecular descriptor name	Index	
8	Hydrophobic moment [55]	34	Distribution of ″MHKFRYW” [51, 52]
9	Distribution of “PATGS” at 0% [51, 52]	35	Distribution of ″ALFCGIVW” at 0% [51, 52]
10	Distribution of “LIFWCMVY” at 0% [51, 52]	36	Distribution of “MPSTHY” at 0% [51, 52]
11	Average sequence composition [56]	37	Distribution of “RKQEND” at 0% [51, 52]
12	Distribution of “HQRKNED” at 25% [51, 52]	38	Distribution of “RKQEND” at 25% [51, 52]
13	Net charge [57]	39	Distribution of “KMHFRYW” [51, 52]
14	Average hydrophilicity [58]	40	Distribution of “DE” [51, 52]
15	Average hydrophobicity CIDH920102 [59]	41	Distribution of “KR” [51, 52]
16	Average hydrophobicity CIDH920104 [59]	42	Distribution of “VIYCWFT” at 50% [51, 52]
17	Average hydrophobicity CIDH920105 [59]	43	Distribution of “ALFCGIVW” [51, 52]
18	Average hydrophobicity MANP780101 [60]	44	Transition from “CLVIMFW” to “RKEDQN” [51, 52]

Index	Molecular descriptor name	Index	
**19**	Average hydrophobicity PONP800105 [61]	**45**	Composition of tripeptide composed by [RKEDQN][GASTPHY][CLVIMFW] [51, 52]
**20**	Distribution of "GASDT" at 75% [51, 52]	**46**	Composition of tripeptide composed by [CLVIMFW][CLVIMFW][GASTPHY] [51, 52]
**21**	Average hydrophobicity PRAM900101 [62]	**47**	Transition from "ALFCGIVW" to "RKEDQN" [51, 52]
**22**	Average hydrophobicity SWER830101 [63]	**48**	Distribution of "GASPHY" at 0% [51, 52]
**23**	Distribution of "GASDT" at 100% [51, 52]	**49**	Distribution of "CLVIMFW" at 0% [51, 52]
**24**	Average hydrophobicity ZIMJ680101 [64]	**50**	Composition of tripeptide composed by [CLVIMFW][CLVIMFW][CLVIMFW] [51, 52]
**25**	Average hydrophobicity WOLR790101 [65]	**51**	Distribution of "GASPHY" at 75% [51, 52]
**26**	Average hydrophobicity CASG920101 [66]		

References

[9] P. Branco *et al.*, "Identification of novel GAPDH-derived antimicrobial peptides secreted by Saccharomyces cerevisiae and involved in wine microbial interactions.," *Applied microbiology and biotechnology*, vol. 98, no. 2, pp. 843–53, Jan. 2014, doi: 10.1007/s00253-013-5411-y.

[30] M. F. da S. Santos, C. S. Freitas, G. C. Verissimo da Costa, P. R. Pereira, and V. M. F. Paschoalin, "Identification of Antibacterial Peptide Candidates Encrypted in Stress-Related and Metabolic

Saccharomyces cerevisiae Proteins," *Pharmaceuticals (Basel)*, vol. 15, no. 2, p. 163, Jan. 2022, doi: 10.3390/ph15020163.

[50] A. Tossi, L. Sandri, and A. Giangaspero, "New consensus hydrophobicity scale extended to non-proteinogenic amino acids - Technische Informationsbibliothek (TIB)," in *European peptide symposium*, pp. 416–417. Accessed: Dec. 12, 2022. [Online]. Available: https://www.tib.eu/en/search/id/BLCP%3ACN054397688/New-consensus-hydrophobicity-scale-extended-to/

[51] K. Tomii and M. Kanehisa, "Analysis of amino acid indices and mutation matrices for sequence comparison and structure prediction of proteins," *Protein Eng*, vol. 9, no. 1, pp. 27–36, Jan. 1996, doi: 10.1093/protein/9.1.27.

[52] Z. R. Li, H. H. Lin, L. Y. Han, L. Jiang, X. Chen, and Y. Z. Chen, "PROFEAT: A web server for computing structural and physicochemical features of proteins and peptides from amino acid sequence," *Nucleic Acids Research*, vol. 34, no. WEB. SERV. ISS., Jul. 2006, doi: 10.1093/nar/gkl305.

[53] L. R. Murphy, A. Wallqvist, and R. M. Levy, "Simplified amino acid alphabets for protein fold recognition and implications for folding," *Protein Eng*, vol. 13, no. 3, pp. 149–152, Mar. 2000, doi: 10.1093/protein/13.3.149.

[54] I. Dubchak, I. Muchnik, S. R. Holbrook, and S. H. Kim, "Prediction of protein folding class using global description of amino acid sequence.," *Proc Natl Acad Sci U S A*, vol. 92, no. 19, pp. 8700–8704, Sep. 1995.

[55] D. Eisenberg, R. M. Weiss, and T. C. Terwilliger, "The hydrophobic moment detects periodicity in protein hydrophobicity.," *Proc Natl Acad Sci U S A*, vol. 81, no. 1, pp. 140–144, Jan. 1984.

[56] P. Klein, M. Kanehisa, and C. DeLisi, "Prediction of protein function from sequence properties. Discriminant analysis of a data base," *Biochim Biophys Acta*, vol. 787, no. 3, pp. 221–226, Jun. 1984, doi: 10.1016/0167-4838(84)90312-1.

[57] M. Charton and B. I. Charton, "The dependence of the Chou-Fasman parameters on amino acid side chain structure," *J Theor Biol*, vol. 102, no. 1, pp. 121–134, May 1983, doi: 10.1016/0022-5193(83)90265-5.

[58] L. A. Kuhn, C. A. Swanson, M. E. Pique, J. A. Tainer, and E. D. Getzoff, "Atomic and residue hydrophilicity in the context of folded protein structures," *Proteins*, vol. 23, no. 4, pp. 536–547, Dec. 1995, doi: 10.1002/prot.340230408.

[59] H. Cid, M. Bunster, M. Canales, and F. Gazitúa, "Hydrophobicity and structural classes in proteins," *Protein Eng*, vol. 5, no. 5, pp. 373–375, Jul. 1992, doi: 10.1093/protein/5.5.373.

[60] P. Manavalan and P. K. Ponnuswamy, "Hydrophobic character of amino acid residues in globular proteins," *Nature*, vol. 275, no. 5681, pp. 673–674, Oct. 1978, doi: 10.1038/275673a0.

[61] P. K. Ponnuswamy, M. Prabhakaran, and P. Manavalan, "Hydrophobic packing and spatial arrangement of amino acid residues in globular proteins," *Biochim Biophys Acta*, vol. 623, no. 2, pp. 301–316, Jun. 1980, doi: 10.1016/0005-2795(80)90258-5.

[62] M. Prabhakaran, "The distribution of physical, chemical and conformational properties in signal and nascent peptides," *Biochem J*, vol. 269, no. 3, pp. 691–696, Aug. 1990, doi: 10.1042/bj2690691.

[63] R. M. Sweet and D. Eisenberg, "Correlation of sequence hydrophobicities measures similarity in three-dimensional protein structure," *J Mol Biol*, vol. 171, no. 4, pp. 479–488, Dec. 1983, doi: 10.1016/0022-2836(83)90041-4.

[64] J. M. Zimmerman, N. Eliezer, and R. Simha, "The characterization of amino acid sequences in proteins by statistical methods," *J Theor Biol*, vol. 21, no. 2, pp. 170–201, Nov. 1968, doi: 10.1016/0022-5193(68)90069-6.
[65] R. V. Wolfenden, P. M. Cullis, and C. C. Southgate, "Water, protein folding, and the genetic code," *Science*, vol. 206, no. 4418, pp. 575–577, Nov. 1979, doi: 10.1126/science.493962.
[66] G. Casari and M. J. Sippl, "Structure-derived hydrophobic potential. Hydrophobic potential derived from X-ray structures of globular proteins is able to identify native folds," *J Mol Biol*, vol. 224, no. 3, pp. 725–732, Apr. 1992, doi: 10.1016/0022-2836(92)90556-y.
